# Exploring Use of Digital Home Assistants for Older Adults: A Demonstration Project

**DOI:** 10.1093/geroni/igab046.1643

**Published:** 2021-12-17

**Authors:** Travis Kadylak, Maya Malecki, Leonardo Galoso, Saahithya Gowrishankar, Amy Brown, Ramavarapu Sreenivas, Wendy Rogers

**Affiliations:** 1 University of Illinois Urbana Champaign, Champaign, Illinois, United States; 2 University of Illinois Urbana-Champaign, Champaign, Illinois, United States; 3 University of Illinois at Urbana-Champaign, Champaign, Illinois, United States; 4 CRIS Healthy Aging Center, Danville, Illinois, United States

## Abstract

Emerging digital home assistant technology has potential to support older adults in their homes. Voice-activated assistants can be used for entertainment, environmental control, physical activities, health management, and social engagement. However, many older adults have limited experience with these devices, which are not designed with them in mind. We conducted a demonstration project to explore how seven older adult assisted and independent living residents interacted with digital assistants over four months. We conducted monthly semi-structured telephone interviews and pre/post questionnaires. Participants desired to use their devices to communicate with others, and for a range of health activities, including nutrition tracking, medication management, and health information searching. However, numerous usability barriers emerged. Some participants perceived their device as a social companion. These findings indicate that older adults are willing to use digital assistants for various activities that may enhance independence, although instructional and training materials are needed to support their use.

